# The kidney, volume homeostasis and osmoregulation in space: current perspective and knowledge gaps

**DOI:** 10.1038/s41526-023-00268-1

**Published:** 2023-04-01

**Authors:** Rik H. G. Olde Engberink, Paula J. van Oosten, Tobias Weber, Kevin Tabury, Sarah Baatout, Keith Siew, Stephen B. Walsh, Giovanna Valenti, Alexander Chouker, Pierre Boutouyrie, Martina Heer, Jens Jordan, Nandu Goswami

**Affiliations:** 1 Amsterdam UMC location University of Amsterdam, Department of Internal Medicine, Section of Nephrology, Meibergdreef 9, Amsterdam, The Netherlands; 2 Amsterdam Cardiovascular Sciences, Microcirculation, Amsterdam, The Netherlands; 3 Space Medicine Team, European Astronaut Centre (EAC), Cologne, Germany; 4 KBR GmbH, Cologne, Germany; 5 Radiobiology Unit, Belgian Nuclear Research Centre, SCK CEN, Mol, Belgium; 6 London Tubular Centre, UCL Department of Renal Medicine, University College London, London, UK; 7 Department of Biosciences, Biotechnologies and Biopharmaceutics, University of Bari Aldo Moro, Bari, Italy; 8 Laboratory of Translational Research Stress and Immunity, Department of Anesthesiology, Hospital of the Ludwig-Maximilians-University (LUM), Munich, Germany; 9 Université Paris Cité, Inserm, PARCC, F-75015 Paris, France; 10 Service de Pharmacologie, DMU CARTE, AP-HP, Hôpital Européen Georges Pompidou, FR-75015 Paris, France; 11 German Aerospace Center (DLR), Institute of Aerospace Medicine, Cologne, Germany; 12 Institute of Nutritional and Food Sciences, University of Bonn, Bonn, Germany; 13 Institute of Aerospace Medicine, German Aerospace Center (DLR) and University of Cologne, Cologne, Germany; 14 Gravitational Physiology and Medicine Research Unit, Division of Physiology, Otto Löwi Research Center of Vascular Biology, Inflammation, and Immunity, Medical University of Graz, Graz, Austria; 15 College of Medicine, Mohammed Bin Rashid University of Medicine and Health Sciences, Dubai, United Arab Emirates

**Keywords:** Physiology, Translational research

## Abstract

Although we have sent humans into space for more than 50 years crucial questions regarding kidney physiology, volume regulation and osmoregulation remain unanswered. The complex interactions between the renin-angiotensin-aldosterone system, the sympathetic nervous system, osmoregulatory responses, glomerular function, tubular function, and environmental factors such as sodium and water intake, motion sickness and ambient temperature make it difficult to establish the exact effect of microgravity and the subsequent fluid shifts and muscle mass loss on these parameters. Unfortunately, not all responses to actual microgravity can be reproduced with head-down tilt bed rest studies, which complicates research on Earth. Better understanding of the effects of microgravity on kidney function, volume regulation and osmoregulation are needed with the advent of long-term deep space missions and planetary surface explorations during which orthostatic intolerance complaints or kidney stone formation can be life-threatening for astronauts. Galactic cosmic radiation may be a new threat to kidney function. In this review, we summarise and highlight the current understandings of the effects of microgravity on kidney function, volume regulation and osmoregulation and discuss knowledge gaps that future studies should address.

## Introduction

The effect of microgravity on kidney function and volume homeostasis has been of interest for researchers since 1966^1^. Fluid redistribution from the legs to the abdomen, thorax and the head was one of the first observations that was considered to impact kidney physiology^2^. Many studies during space flight and simulated microgravity on Earth have tried to establish the effects of microgravity on kidney function and volume homeostasis. However, essential questions remain unanswered.

Investigation of kidney physiology in space is complex because of the simultaneous changes in osmoregulation, volume regulation and glomerular and tubular function in response to microgravity. To understand the exact impact of microgravity, all responses should be thoroughly tested. This goal is difficult to achieve due to logistic issues and the fact that most measurements are influenced by fluid and nutrient intake, countermeasures, and space flight duration.

Better understanding of kidney physiology and volume homeostasis in space is of special interest because orthostatic intolerance commonly occurs after space flight, which is still unresolved despite the use of extensive countermeasures^3^. More targeted countermeasures that are directed to intervene in kidney physiology and volume homeostasis could conceivably ameliorate orthostatic intolerance. The problem of orthostatic intolerance will become even more important during future exploration missions when astronauts are exposed to longer periods of microgravity and then get re-exposed to a hypogravity environment upon landing on a planetary surface without the usual medical care that is available after return to Earth^4^. In these situations, an orthostatic intolerance-induced fall could be life- and mission threatening. In addition, kidney stone formation could jeopardize future long-term deep space missions where evacuations times will be significantly longer than currently from the International Space Station. In this review, we discuss the key findings of the last decades of relevant research and highlight the most important research gaps concerning volume regulation, osmoregulation and kidney function that should be addressed to prepare astronauts and space medicine experts for future deep space exploration missions (Table 1).

**Table 1:** Research gaps concerning kidney function, volume homeostasis and osmoregulation in space.

**Sodium and water balance**
Short and long-term sodium balance
Long-term rhythms of aldosterone and cortisol
Effect of aldosterone and cortisol rhythms on sodium balance
Effects of sex hormones on sodium balance
Skin and muscle sodium content, and its hemodynamic effects
Relation between skin water loss and skin sodium content
Long-term rhythms of antidiuretic hormones
Relation between muscle loss, urea generation and catabolism
Effects of muscle retention on muscle sodium content
Effects of sodium, water and protein intake on skin sodium content
Effects of sodium, water and protein intake on muscle mass and renal sodium wasting
Differences in sodium and water balance during space travel and bed rest
**Glomerular and tubular function**
Microgravity and sodium and chloride transporters
Composition of kidney stones before, during and after space flight
Prevalence and severity of hypercalciuria
The potential connection between bone loss and hypercalciuria
Pharmacological and dietary intervention to limit hypercalciuria
Effect of vitamin D supplements on calciuria and kidney stones
Effect of lunar/Martian dust on glomerular and tubular function

## Volume regulation

### Body weight and compartments

The central redistribution of fluids was thought to be the main factor impacting body fluid regulation in space. However, some space flight observations cannot be explained by this mechanism. For many years, the immediate and persistent loss of body weight was attributed to fluid loss secondary to increased diuresis as a result of the Henry-Gauer reflex^5^. However, further research demonstrated that diuresis early inflight did not take place, total body water content did not change, and that the reduction of extracellular and plasma volume is compensated by an increase in intracellular volume during short-term space flight^6^. In line with this observation, Leach et al. did not find any increase in sodium or water excretion that could induce a reduction in total body water. Similar responses were observed in head-down tilt bed rest studies^7–9^.

### Sodium homeostasis and tissue sodium storage

**Fig. 1:**
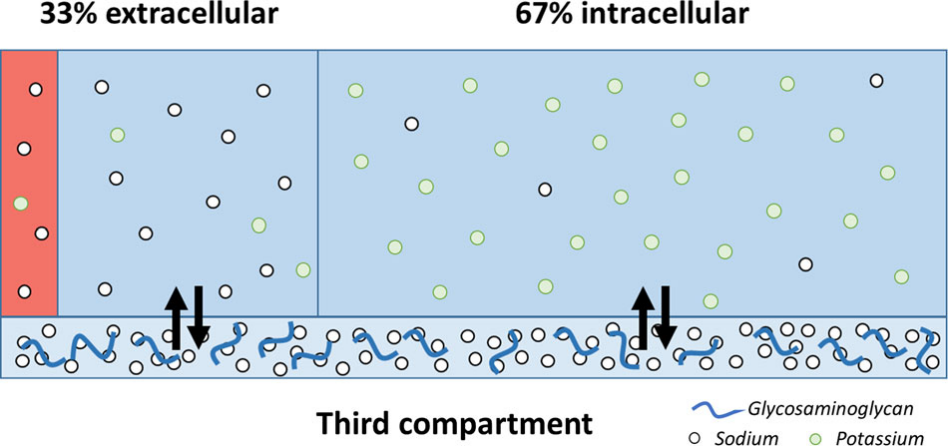
Body fluid composition on Earth. High sodium concentrations (~140 mmol/L) and low potassium concentrations (~4 mmol/L) are found in the extracellular compartment, which consists of an interstitial (blue) and intravascular (red) space. In the intracellular compartment, which compromises 67% of total body water, sodium concentration is low (~10 mmol/L) and potassium concentrations are high (140–150 mmol/L). The classical view on sodium homeostasis suggests that all compartment are equilibrated and that changes in extracellular sodium concentration induce a fluid shift from the intracellular to extracellular compartment. However, supraphysiological high sodium concentrations have been found in the skin and muscle, constituting a third compartment, which is not in osmotic equilibration with the other fluid compartments^98^.

Recently, there has been a paradigm shift in sodium and water homeostasis, which may shed new light on sodium balance during space flight. Long-term balance studies and metabolic ward studies demonstrated that sodium homeostasis is more complicated than the established intra- and extracellular compartment model^10–12^. These studies showed that 24-hour sodium excretion can differ up to 80 mmol from 24-hour sodium intake during fixed sodium intake, thereby inducing large fluctuations in total body sodium content up to thousands of mmols over weeks (Fig. 1). Surprisingly, these substantial fluctuations in total body sodium content, which were related to infradian cortisol and aldosterone rhythms, did not result in concomitant changes in body water, body weight, or blood pressure, and were associated with low-grade metabolic acidosis^10,12,13^. These data challenge previous assumptions on sodium homeostasis of the past 70 years.

Experimental studies suggest that sodium excess can be neutralized by negatively-charged glycosaminoglycans in the skin, skeletal muscles, and glycocalyx^14^. After binding to glycosaminoglycans, sodium is osmotically inactivated such that sodium retention is not accompanied by water retention. As a result, local sodium concentrations can increase significantly. This process has been described as nonosmotic sodium storage (Fig. 1). Others demonstrated that excess sodium may be shifted to the intracellular compartment, in particular to skeletal muscle^15^. In fact, high skin and muscle sodium content has been observed in patients with arterial hypertension, hyperaldosteronism and heart failure, and after high sodium intake^16,17^.

The exact cause and function of skin sodium accumulation in humans is not yet understood but experimental evidence demonstrated involvement of the lymphatic system and immune cells, in particular macrophages, in this process^18^. More recently, increased skin sodium content was hypothesized to be an evolutionary measure to limit skin water loss and conserve water in situations of water loss or shortage, which could come at the expense of an increase in blood pressure and muscle catabolism^19^.

### Tissue sodium storage during space flight

**Fig. 2:**
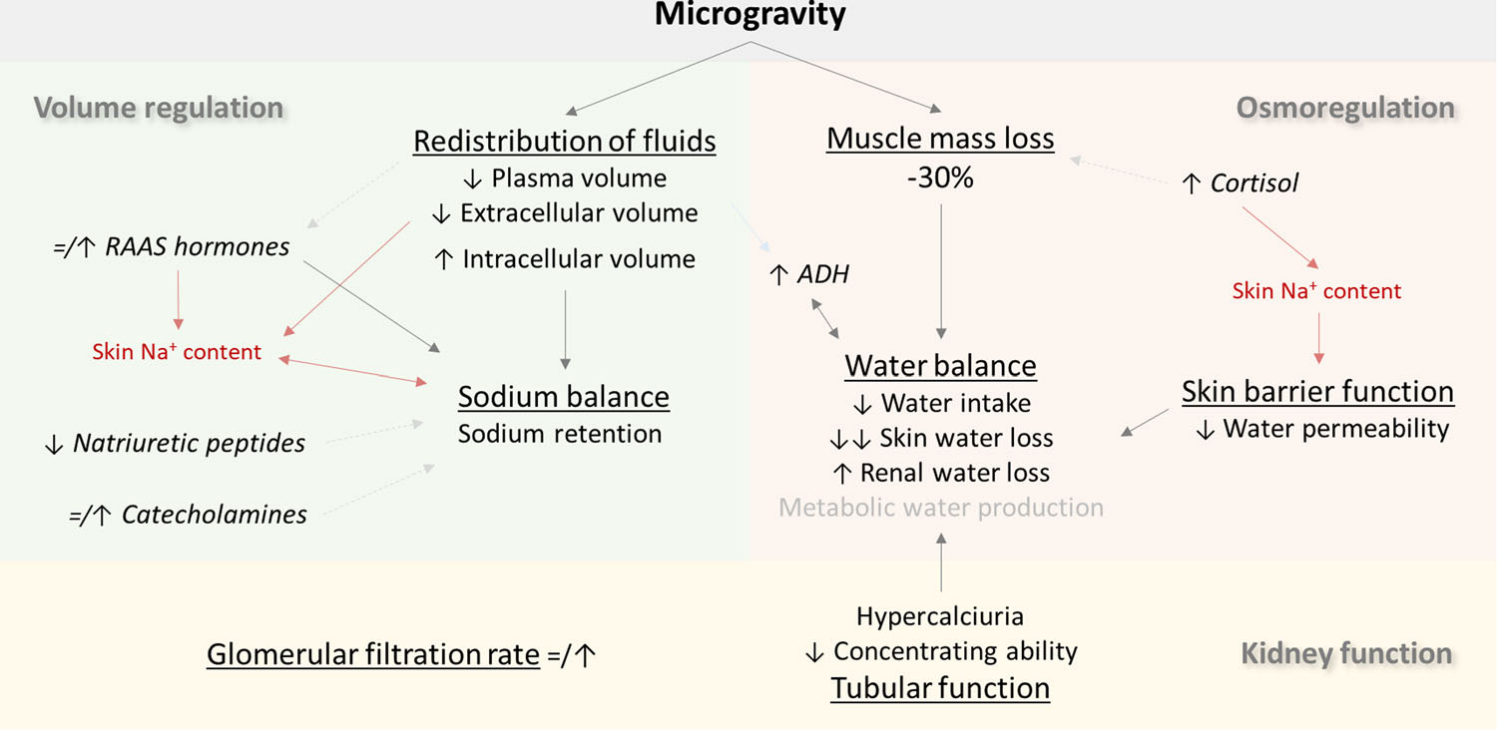
Hypothesized effects of microgravity on kidney function, volume and osmoregulation. Light grey lines and text indicate findings that are not consistent in literature or have not been investigated thoroughly. Lines in red depict the hypothesized effect of sodium storage in the skin and muscle compartment. ADH antidiuretics hormone, RAAS renin-angiotensin-aldosterone system.

The discovery of a third compartment for sodium accumulation resulted in new insights for common problems such as hypertension and hyponatremia, but it may also affect hemodynamics and osmoregulation in space (Fig. 2)^20–22^. The space flight observation that plasma volume and blood pressure decrease while heart rate increases could be explained in part by volume depletion^7,23^. However, a high sodium diet during head-down tilt bed rest did not reverse the decrease in plasma volume, but even resulted in a greater loss of plasma volume, and data from space flight experiments demonstrated significant sodium retention^5,8,9,24,25^.

These findings challenge the traditional concept that ingested sodium is either added to the extracellular space, thereby restoring plasma and interstitial volume, or excreted, thereby stabilizing sodium balance. Some of the ingested sodium seems to disappear without inducing the expected fluid retention^24^. These data suggest that the ‘missing’ sodium is stored in the third compartment, which is not in equilibrium with plasma volume and prevents sodium from being excreted by the kidneys, or could be moved to the intracellular compartment.

So far, tissue sodium storage has not been investigated during space travel or bed rest studies. Also, it is unknown how much sodium can potentially accumulate in this third compartment during bed rest. A previous study reported an increasing total body sodium content from day 2 to the study end at day 18 resulting in a 741-mmol positive sodium balance^24^. The amount of sodium that can accumulate in the third compartment is particularly relevant for future deep space exploration missions with long exposure times to microgravity and subsequent planetary surface exploration activities.

**Fig. 3:**
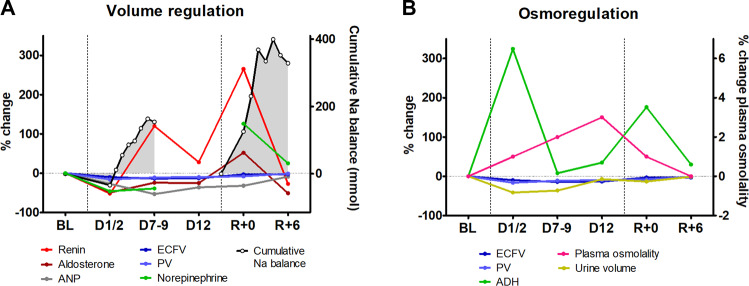
Interaction among the neurohumoral system and sodium and water balance. Data on volume regulation (**A**) and osmoregulation (**B**) responses, which are derived from the 9-day and 14-day Spacelab Life Sciences 1 and 2 (SLS-1 and SLS-2) missions^6^. The cumulative sodium balance data is reset at the end of space flight (before R + 0). ADH antidiuretic hormone, ANP atrial natriuretic peptides, BL pre-flight baseline, D day(s) into space flight, R day after return to Earth, ECFV extracellular fluid volume, Na sodium, PV plasma volume.

During space flight, the third compartment for sodium accumulation (i.e. skin interstitium and muscle) is subject to change. Most studies investigating extracellular volume have reported an initial and persistent reduction without restoration of extracellular volume to pre-existent values, but data on the skin compartment, which represents an important part of the extracellular volume, are scarce^26^. The most comprehensive study on skin function during space flights suggests that skin thickness is not affected by microgravity but other studies have reported opposite results^27^. Contrary to the above, the muscle compartment has been extensively investigated: muscle volume has been shown to decrease, with up to 30% loss of muscle volume in certain muscle groups of the lower, weight-bearing limbs^28^. The reduction of muscle volume could potentially decrease the capacity for muscle sodium accumulation. One may hypothesize that this may contribute to orthostatic intolerance after space flight by reducing the ability to release sodium from the tissues when gravity is restored. This hypothesis is in line with a recent study showing a release of sodium from the tissue stores within 30 min after a hypotonic stimulus^20^. It is, however, unknown whether tissue sodium may be able to play a role in acute hypovolemia. Interestingly, even after return to earth, the body keeps on retaining sodium during the first days (Fig. 3)^6^.

Muscle sodium storage capacity may be restored by countermeasures that are directed to prevent muscle atrophy. This hypothesis is supported by recent studies demonstrating that the combination of exercise training during space travel and saline loading right after space travel is able to mitigate orthostatic related symptoms^29,30^. The exact contribution of both interventions to the mitigation of orthostatic hypotension and potential restoration of the nonosmotic sodium storage capacity is unknown.

Considering the recently proposed role of cortisol in sodium homeostasis, one could hypothesize that the hypothalamic-pituitary-adrenal axis could contribute to volume regulation during microgravity. Throughout the relatively short Skylab flights, cortisol levels were increased but long-duration space flights showed that cortisol levels return to baseline values after 160 days^6,31,32^. High cortisol levels are generally known to impact protein and muscle catabolism but the recently discovered role in infradian sodium excretion rhythms is less known^10^. Although cortisol can bind to the mineralocorticoid receptors, thus promoting sodium retention, long-term balance studies associated high cortisol levels with a negative sodium balance^10,33^. Future studies are needed to investigate the interaction between cortisol, (muscle) catabolism and long-term volume and osmoregulation during space flight.

### Renin, aldosterone and natriuretic peptides

The renin-angiotensin-aldosterone system has been studied many times during space flight with different results. These conflicting data can, at least in part, be explained by differences in assays, body posture and space flight duration. Overall, renin and aldosterone levels sharply decrease on the first day of space flight and seem to return to or above pre-flight values thereafter^6,25,34^. Like on Earth, a high sodium diet or sodium infusion are able to decrease aldosterone levels indicating that sodium balance remains linked to the renin-angiotensin-aldosterone system (RAAS)^25,35^. Nevertheless, RAAS hormones are not always in line with the net sodium balance, in particular as observed during early space flight when a positive sodium balance was observed and aldosterone levels were low to normal^6^.

Natriuretic peptides also fall right after space flight but, in contrast to RAAS hormones, remain significantly suppressed after four weeks in space^6,25^. Mid-regional pro-atrial natriuretic peptide responded similarly to an increase in sodium intake from 2 to 5.5 grams/day in space and on Earth, however, measurements were reset to lower levels in space^25^. This finding may be partly explained by the reduction in thoracic blood volume during space flight^25^. So far it is unknown whether the reduction in natriuretic peptides is an appropriate physiological response to reduced cardiac preload or whether the response is maladaptive such that sodium balance gets out of tune.

### Sympathetic nervous system

Data on sympathetic nervous activity during space flight are contrasting and may be related to mission duration as well as inter-individual variability in the susceptibility to the space environment^36^. Several studies demonstrated that plasma and urine catecholamine levels do not change during long-term space flight^37,38^. On the other hand, measurements of whole-body noradrenaline spill-over and peroneal nerve muscle sympathetic activity in six male astronauts during a shorter term space shuttle mission indicated increased sympathetic activity^39^. Possibly, sympathetic measurements during shorter term missions may be confounded by the psychological and physiological stresses imposed by adaptation to the space environment combined with an intense work schedule. Likewise, data after return to Earth show variable sympathetic responses. Although two studies observed a sharp increase in sympathetic activity, a third study could not reproduce this finding despite a substantial increase in cardiac output and decrease in blood pressure^37,38,40^. However, the link between orthostatic intolerance and sympathetic nerve activity is supported by studies that relate orthostatic complaints to an attenuated increase in plasma norepinephrine and increased norepinephrine metabolites after space flight, and increased plasma norepinephrine after bed rest^23,41,42^. Interestingly, plasma norepinephrine levels were not affected by acute saline infusion suggesting that a change in volume status may not be the sole mechanism affecting sympathetic nervous activity during spaceflight^35^. Given the importance of the sympathetic nervous system in maintaining orthostatic tolerance and the intense cross-talk between sympathetic nervous system and the RAAS system, which is reviewed in the following section, there is a need for additional studies on sympathetic responses during long duration missions.

### Interactions between the sympathetic system and RAAS hormones

Sympathetic activation is one of the driving forces of the renin angiotensin system, either renal, tissular or brain^43^. Both sympathetic activation and RAAS activation may lead to sodium retention, and contribute to the variability in blood pressure responses during space flight^44^. However, only two studies with ten subjects on three different space flights investigated the interaction between neurohumoral systems and sodium and water balance (Fig. 3)^6^. The overstimulation of the sympathetic system and RAAS during spaceflight contrasts with the lower levels of cardiac natriuretic peptides^45^. On Earth, cardiac natriuretic peptides and the renin-angiotensin-aldosterone axis are usually regulated in a reciprocal fashion. Whether this resetting of natriuretic peptides in space reflects appropriate responses to reduced central blood volume, true natriuretic peptide deficiency or dysregulation of the RAAS or sympathetic system deserves further studies. These questions are worth investigating since cardiovascular drugs targeting these systems have transformed the prognosis of hypertension and heart failure by treating excessive counter regulations rather than the original cause of disease. In many instances, adaptation to spaceflight might resemble this situation, and cardiovascular drugs targeting neurohormonal response represent interesting candidate drugs for prevention of spaceflight-induced health issues.

## Osmoregulation

### Water balance

Microgravity influences water balance but the benefits or mechanisms behind these changes are poorly understood. The available data on water balance should be interpreted in the light that all more recent spaceflights required a minimal oral water intake after the observation that fluid intake decreased significantly when astronauts were not reminded of drinking during the first space missions^34,46,47^. Taking this and other limitations of space flight studies into account, Drummer et al. concluded that the net water balance during microgravity exposure is likely to be neutral^5^. Nevertheless, water balance is subject to substantial changes in thirst sensation as well as renal and skin water losses.

The reduction in fluid intake upon space travel may result from decreased thirst sensation^48^. This observation cannot be explained by hypo-osmolality, increased extracellular volume or high levels of natriuretic peptides, three triggers that are known to lower thirst sensation on Earth^49,50^. Possibly, lower angiotensin-II levels, which may be anticipated from the reduced renin levels during early space flight^6^, may contribute to reduced thirst sensation, although angiotensin-II has been mainly demonstrated to *increase* thirst upon extracellular volume depletion or infusion, and data on an acute decrease in angiotensin-II levels are not available to this date^50^. Also, thirst sensation could be less due to motion sickness but this could only explain the reduced thirst sensation during the first days upon arrival in microgravity as space motion sickness typically resolves itself after a couple of days^51^.

An alternative explanation of diminished fluid intake may be the production of endogenous metabolic water. Recently, data derived from a terrestrial space station simulating a Mars journey could demonstrate that body water regulation and energy homeostasis are intimately linked by a fundamental physiological adaptation principle designed to prevent dehydration^52,53^. These studies related muscle catabolism, which is a major problem during space travel, to metabolic water production. To this date, this aestivation-like mechanism, which has been shown to be activated during renal or skin water loss, has not been explored in microgravity^19,54^.

Another determinant of water balance is skin water loss. In contrast to what would be expected in hypobaric conditions, transepidermal water loss is reduced during space flight by approximately 10–20%^27,47^. This is accompanied by an improved skin hydration status^27^. An intervention study that temporarily increased skin temperature by 1° Celsius found that, after bed rest, there was an increased threshold for cutaneous vasodilation and sweating^55^. These data on skin barrier function during space flight parallel the findings of a recent study in rats with chronic renal failure and secondary muscle catabolism, increased skin sodium content and reduced skin water loss^19^. This experimental study suggested that a reduction in skin water loss may reflect an adaptive response to overcome excessive (renal) fluid losses and is associated with high skin sodium content.

Data from studies with fluid intake interventions may help to understand the physiology of water balance. Sato et al. conducted a 20-day study of head-down tilt bed rest and instructed participants to drink the amount of fluid that was excreted by the kidneys the previous day^56^. This approach resulted in subjects drinking and urinating more and more every day, from approximately 1100 ml/day to 2400 ml/day at the end of the study. These data suggest that there is a second source of water, which may be of metabolic origin due to muscle wasting, and that the reduced fluid intake may be a consequence. Another study investigated an oral water load of 600 mL during short- and long-term space flight and bed rest. Unexpectedly, urinary flow rate did not increase in space whereas a 5-fold increase was observed on Earth after acute supine position, short-term or long-term bed rest^57^. The observations in space suggest dehydration at baseline resulting in water retention but measurements of urine output and osmolality, after an overnight fast, were similar to the bed rest groups and did not indicate dehydration. This study underscores the lack of understanding of the water balance during microgravity and the notion that data from bed rest studies can be significantly different from data during actual space flight^58^.

### Antidiuretic hormone

Water balance is mainly regulated by antidiuretic hormone (ADH), which is secreted upon high plasma osmolality or decreased extracellular volume. During long-term space flight, ADH levels are increased^5,48^. As plasma osmolality does not change after long-term space flight, the increased levels may be the consequences of decreased plasma and extracellular volume. Grigoriev et al. showed that the antidiuretic effect of ADH may be attenuated as a result of spaceflight, by demonstrating that higher ADH levels were needed to achieve a similar reabsorption rate of osmotically free water^48^. An important limitation, however, is that measurements were performed directly post-flight, after restoration of gravity. Other post-flight measurements show that the urine concentrating ability of the kidney after a 12-hour food and water deprivation test decreases with space flight to a maximum urine osmolality of 782 mOsm/L after 3–6 months of space flight, which could also be explained by a limited capacity to increase the tonicity of the renal medulla.

In the previously discussed water loading study of Norsk et al., which demonstrated an attenuated increase in urinary flow rate after water loading during space flight compared to bed rest, ADH was not measured^57^. However, the limited decrease in urine osmolality after water loading during space flight suggests either increased ADH levels or changes in the responsiveness of renal collecting ducts to ADH^57^. This discrepancy between space flight and bed rest may be explained by the observations that, in contrast to the increased ADH levels during space flight, ADH decreased during bed rest^59,60^.

## Kidney function

### Glomerular function

Reports on the effects of space flight on glomerular function vary. Leach et al., who measured creatinine and inulin clearance simultaneously during space flight, reported an increase during the first days and normalization to baseline values afterwards^6^. Surprisingly, this increase took place during the initial reduction in extracellular and plasma volume, and reduced fluid intake, although the latter has been demonstrated to increase glomerular filtration rate on Earth^61^. Another remarkable observation is that creatinine clearance, which is known to overestimate glomerular function, was lower than measured inulin clearance. The observed reduction in plasma urea concentration supports the temporary increase in glomerular filtration rate^6^.

The transient increase in (estimated) glomerular filtration rate has not been observed in older studies, which can be explained by the lack of in-flight measurements and the absence of instructions to maintain adequate fluid intake in these studies.

The observed increased glomerular filtration rate by Leach et al. was not associated with a significant increase in effective renal plasma flow, which suggests an increase in filtration fraction^6^. This finding cannot explain the previously discussed observation of sodium retention, which may therefore be of tubular origin. On the other hand, the large standard deviation of effective renal plasma flow should be taken into account. The coefficient of variation of day-8 creatinine clearance and effective renal plasma flow in the study of Leach et al. was 27% and 39%, respectively, whereas the percentage change from baseline was 20% and 17%, respectively^6^. This suggests that the low sample size and the relative inaccuracy of effective renal plasma flow measurements may contribute to the previously suggested difference in both variables. An alternative explanation is that space conditions led to a change in the balance between afferent and efferent arteriolar constriction in renal glomeruli thereby increasing filtration fraction.

During bed rest, in healthy men, plasma creatinine levels gradually decreased to a new steady state after 2–3 weeks reflecting loss of muscle mass^62^. As 24-hour creatinine excretion did not change this indicates an increase in creatinine clearance in the first 2–3 weeks after bed rest. Interestingly, cystatine C levels showed a similar decrease as plasma creatinine. Contrary to space flight observations, plasma urea increased during the first weeks of bed rest and was related to the observed reduction in muscle mass^62^. Interestingly, Arinell et al. found different results in females^63^. After 60 days of bed rest, plasma cystatin C showed a significant increase whereas creatinine levels remained stable. These data underline the importance that future studies should take sex-specific effects into account.

### Tubular function

Histopathological assessment of rat kidneys after simulated microgravity demonstrate degeneration and necrosis of tubular epithelial cells^64^. One could expect that such changes would translate into proteinuria, impaired urine acidification, altered urine electrolyte content or a diminished renal concentrating ability.

The most notable change in 24-hour urine composition during space flight is the increase in urine calcium excretion, which is attributed to increased bone mineral loss. Daily urine calcium excretion has been reported to increase by approximately 20–80 mg/day during microgravity and bed rest^65–67^.

Hypercalciuria has been linked to water balance as high urine calcium content has been demonstrated to induce renal water loss by proteolysis of aquaporin-2 and thereby limiting the maximal urinary concentration and calcium saturation^68–70^. The amount of water loss due to hypercalciuria is likely to be clinically relevant. In hypercalciuric children, hypercalciuria was associated with a 250-mL higher 24-hour urine volume and a 147-mOsm/kg lower urine osmolality^70^. Administration of synthetic ADH (DDAVP) in these hypercalciuric children increased urine osmolality, but only to the baseline levels of normocalciuric children. In bed rest studies, a decrease in aquaporin-2 excretion was associated with increased calcium excretion supporting the view that urinary calcium can modulate ADH-dependent urine concentration through down-regulation of aquaporin-2 expression/trafficking^68^. This phenomenon could have a key role in the prevention of urine supersaturation and stone formation due to hypercalciuria and may contribute to the uncoupling of sodium and water balance during space flight.

Pastushkova et al. analysed urine protein composition before and after space flight. Three proteins, which were not found in the urine on Earth, could be detected in the urine after space flight^71^. One of these proteins, aminopeptidase A, is a marker of renal tissue hypoxia and tubular dysfunction. However, aminopeptidase A was only found seven days after space flight and was not observed three days after return to Earth. Given the lack of in-flight measurements, this may therefore represent changes after space flight instead of changes induced by space flight. Future studies investigating the effects of tubular function during microgravity should therefore focus on in-flight measurements.

### Kidney stones

Astronauts and cosmonauts have an unusually high rate of kidney stone formation, despite being screened for absence of kidney stone history. Stones are usually seen after space flight^67^. Among 357 astronauts, 22 experienced at least one episode of kidney stones with a total of 36 episodes^72^. Kidney stone formation is of mission critical significance. In the past, one Soviet in-flight renal stone episode nearly caused a mission termination due to intractable symptoms, but was relieved by spontaneous stone passage by the cosmonaut just before an urgent deorbit was initiated^73^. It has been demonstrated that space flight associated changes in urinary biochemistry favour kidney stone formation^74,75^. Besides hypercalciuria due to bone mineral loss, hyperphosphaturia, hypocitraturia and a decrease in urine pH have been reported following microgravity^67^. These changes are the consequence of the high dietary acid load whereas phosphate is also from bone origin^76^. In addition, the reduced water intake and diuresis is a major risk factor for kidney stones. Interestingly, microgravity may also have a direct effect on the crystallisation and nucleation of nascent kidney stones, but this is dwarfed by the net biochemical urinary changes observed in space flight^77^. Changes in the microarchitecture of the kidney can lead to abnormal renal calcification and stones (e.g. in medullary sponge kidney), and changes in the nephron architecture can occur with the kinds of electrolyte disturbance that can be caused by space flight^78,79^.

A randomized, double-blind, placebo-controlled study that was performed during space flight demonstrated that supplementation of potassium-citrate lowers urinary calcium excretion and increases urine citrate levels and urine pH during space flight thus reducing the propensity to cristallization^65^. For this reason, potassium-citrate is routinely used by crewmembers who are felt to be at elevated risk for stone formation. During bed rest, potassium-magnesium-citrate supplements were demonstrated to attenuate the increase in calcium excretion and increase in urine pH, potassium, magnesium and citrate levels^66^. Besides these supplementations, compliance with the daily recommended fluid intake of >2 litre is crucial to lower the risk for renal stone formation. Other dietary recommendation such as a low protein and sodium diet, which are advised to individuals at risk for renal stones on Earth, are thought to have a similar beneficial effect during space flight but hard data to support these recommendations are lacking. Considering the previously discussed knowledge gaps on sodium and water homeostasis and muscle metabolism, the exact net effect of these general recommendations may turn out differently.

By interfering with bone resorption, the renal excretion of calcium may also be limited. Administration of pamidronate was shown to reduce 24-hour calcium excretion with 55–65% during the first 30 days of bed rest whereas resistive exercise training was not effective^80^. These large effects, however, could not be reproduced during space flight^26^. A more recent study demonstrated that the combination of alendronate and advanced resistive exercise device training could lower daily calcium excretion below pre-flight values and prevent an increase in calcium oxalate and calcium phosphate relative supersaturation, an effect that could not be achieved with exercise only^81^. In the last decades, vitamin D supplementation was initiated during space flight and doses were increased to 1000 IU per day, which was demonstrated to keep vitamin D at adequate levels without any adverse effects^82^. However, the exact relation between vitamin D dose, 24-hour calcium excretion and renal stone risk during space flights remains to be defined.

### Galactic cosmic radiation

There is concern regarding the effect of galactic cosmic radiation (GCR) exposure on the longer missions planned as part of the Artemis Program and the Deep Space Transport/Mars Missions. To date this has mostly focused on the risk of carcinogenesis but the kidney is an exquisitely radiation sensitive organ^83–85^. In fact, the kidney is the dose limiting organ in abdominal radiotherapy and total body irradiation^83^. Beside mitochondrial damage due to the oxidative stress generated by gravitational changes, GCR can further enhance mitochondrial damage through the radiation-induced reactive oxygen species^86^. The resulting mitochondrial damage affect the kidneys at multiple levels in both tubular and glomerular integrity, and micro- and macrovascular function^87,88^. To this date, data are limited, but there is emerging animal and astronaut data that link exposure to high atomic number and energy (HZE) ions to mitochondrial damage^89,90^. We postulate that the most vulnerable component of the kidney architecture is the proximal tubule, because of its high oxidative metabolism (mitochondria rich), its low glycolytic ability and its crucial role in mass transport of solutes and water. Indeed, tubular injury is an early and prominent histological feature in iatrogenic radiation nephropathy^91^. Due to their full dependence on mitochondrial aerobic metabolism, proximal tubular cells are extremely vulnerable to mitochondrial damage. Altogether, our understanding on the molecular and cellular mechanisms linked to how chronic exposure to GCR impacts kidneys remains to be elucidate and warrant urgent investigation to develop mitigation strategies.

### Clinical pharmacology and the kidney

The kidney is not only an essential organ for fluid and ions homeostasis, it is also the main excretion pathway for xenobiotics, among them drugs and toxic compounds. Systematic pharmacodynamic and pharmacokinetic studies in space are lacking. Yet, the profound change in renal physiology could conceivably affect renal drug elimination. Moreover, anecdotal evidence suggests that the response to drug therapies may be altered in space. This issue coupled with limited shelf life of many medications and potential degradation through radiation might be particularly problematic during long-term lunar or mars missions where it is not possible to return to Earth on short notice^92^. To date, around 80 different drugs are available on the ISS^93^. Yet, only the pharmacokinetics of acetaminophen, scopolamine, promethazine and antipyrine have been tested in space^94^. Although, these studies have several limitations, an apparent trend toward altered drug disposition during spaceflight compared to measurements on Earth could be seen^92,94^. In particular, the expression changes of CYP450 superfamily of enzymes, which metabolizes 70–80% of all prescription drugs, was observed in rodents, but data in humans are lacking^93,95^. Drugs acting on the kidney might be useful for prevention of lithiasis and bone loss. For instance, thiazide-like compounds decrease calciuria, lower the incidence of recurrent kidney stones, prevent bone loss and appear to prevent hip fractures, but their use is limited by the lack of knowledge about their pharmacodynamics and kinetics during spaceflight^96,97^. Better understanding of renal changes and their influence on blood detoxification is a call for interdisciplinary programs.

## Outlook and summary

To be able to safely increase the length of future space missions, we need future studies to investigate basic kidney physiology, osmoregulation and volume regulation, and develop new countermeasures that are directed to preserve kidney function and prevent potential life-threatening orthostatic complaints and kidney stone formation. A substantial part of these data could be gathered in studies that investigate other hypotheses but allow accurate documentation of sodium and water balance, and repeated urine collections and blood draws for relatively simple analyses of RAAS and antidiuretic hormones, natriuretic peptides, sympathetic nervous activity and catabolic hormones.
