# Author Correction: The kidney, volume homeostasis and osmoregulation in space: current perspective and knowledge gaps

**DOI:** 10.1038/s41526-023-00307-x

**Published:** 2023-08-01

**Authors:** Rik H. G. Olde Engberink, Paula J. van Oosten, Tobias Weber, Kevin Tabury, Sarah Baatout, Keith Siew, Stephen B. Walsh, Giovanna Valenti, Alexander Chouker, Pierre Boutouyrie, Martina Heer, Jens Jordan, Nandu Goswami

**Affiliations:** 1 Department of Internal Medicine, Section of Nephrology, Amsterdam UMC location University of Amsterdam, Meibergdreef 9, Amsterdam, The Netherlands; 2 Amsterdam Cardiovascular Sciences, Microcirculation, Amsterdam, The Netherlands; 3 Space Medicine Team, European Astronaut Centre (EAC), Cologne, Germany; 4 KBR GmbH, Cologne, Germany; 5 Radiobiology Unit, Belgian Nuclear Research Centre, SCK CEN, Mol, Belgium; 6 London Tubular Centre, UCL Department of Renal Medicine, University College London, London, UK; 7 Department of Biosciences, Biotechnologies and Biopharmaceutics, University of Bari Aldo Moro, Bari, Italy; 8 Laboratory of Translational Research Stress and Immunity, Department of Anesthesiology, Hospital of the Ludwig-Maximilians-University (LUM), Munich, Germany; 9 Université Paris Cité, Inserm, PARCC, F-75015 Paris, France; 10 Service de Pharmacologie, DMU CARTE, AP-HP, Hôpital Européen Georges Pompidou, FR-75015 Paris, France; 11 German Aerospace Center (DLR), Institute of Aerospace Medicine, Cologne, Germany; 12 Institute of Nutritional and Food Sciences, University of Bonn, Bonn, Germany; 13 Institute of Aerospace Medicine, German Aerospace Center (DLR) and University of Cologne, Cologne, Germany; 14 Gravitational Physiology and Medicine Research Unit, Division of Physiology, Otto Löwi Research Center of Vascular Biology, Inflammation, and Immunity, Medical University of Graz, Graz, Austria; 15 College of Medicine, Mohammed Bin Rashid University of Medicine and Health Sciences, Dubai, United Arab Emirates

**Keywords:** Physiology, Translational research

Correction to: *npj Microgravity* 10.1038/s41526-023-00268-1, published online 01 April 2023

The original version of this Article omitted the authors Keith Siew and Stephen B. Walsh from the London Tubular Centre, UCL Department of Renal Medicine, University College London, London, UK.

Consequently, the following was originally omitted from the Acknowledgements: ‘K.S. is supported by the Wellcome Trust [110282/Z/15/Z]. K.S. and S.B.W. are supported by the UK Space Agency [ST/X000036/1]’.

Additionally, the following was originally omitted from the Author Contributions: ‘T.W., K.T., S.B., K.S., S.W., G.V., A.C., P.B., M.H., J.J. and N.G. critically revised the manuscript. All authors reviewed and approved the final version of the manuscript.’

This has been corrected in both the PDF and HTML versions of the article.

